# Effect of filament types and loops number on the force degradation of elastomeric chains used for orthodontic treatment: an in-vitro study

**DOI:** 10.1186/s12903-023-02812-7

**Published:** 2023-02-19

**Authors:** Bowen Zheng, Majedh Abdo Ali Al-Somairi, Zhiyuan Li, Yang Zhao, Yi Liu

**Affiliations:** 1 Department of Orthodontics, School and Hospital of Stomatology, China Medical University, Liaoning Provincial Key Laboratory of Oral Diseases, Shenyang, 110002 China; 2 Department of Orthodontics and Dentofacial Orthopedics, Faculty of Dentistry, Ibb University, Ibb, Republic of Yemen

**Keywords:** Elastomeric chain, Force degradation, Thermal cycle

## Abstract

**Background:**

In orthodontic treatment, closing spaces, specifically the extraction and scattered spaces of the anterior teeth, requires some auxiliary bias, such as an elastomeric chain. Many factors affect the mechanical properties of elastic chains. In this study, we investigated the relationship of the filament type, the number of loops, and the force degradation of elastomeric chains under thermal cycling conditions.

**Methods:**

The orthogonal design included three filament types (i.e., close, medium, and long). Four, five, and six loops of each elastomeric chain were stretched to have an initial force of 250 g in an artificial saliva environment at 37 °C and thermocycling between 5 and 55 °C three times a day. The remaining force of the elastomeric chains was recorded at different time points (4 h, 24 h, 7 days, 14 days, 21 days, and 28 days), and the percentage of the remaining force was calculated.

**Results:**

The force decreased significantly in the initial 4 h and degraded mostly within the first 24 h. In addition, the percentage of force degradation increased slightly between 1 and 28 days.

**Conclusions:**

Under the same initial force, the longer the connecting body is, the fewer the number of loops and the greater the force degradation of the elastomeric chain are.

## Background

Elastomeric chains have long been popular in the orthodontic field [1]. They are extensively used for intra-arch teeth movement and force transmission [2]. Orthodontists have used these chains because they are practical, convenient, efficient, affordable, low cost, comfortable for patients, and effective in closing spaces by applying light continuous orthodontic forces, which are preferable to produce dentoskeletal alterations [3–9]. Elastomeric chains, as an adjunct traction device [10], were first introduced in orthodontics at the end of the 1960s not only for canine distal movement, the closure of the extraction space, and the scattered space of the anterior teeth but also for the correction of reversing teeth [11–13].

Elastomeric chains come in three forms: short (open), long (wide), and closed (continuous). They can also be transparent, gray, or a different color depending on their specific properties and intended use [14]. Orthodontic manufacturers may keep their exact composition secret for various reasons [14, 15]. The elastomeric chain is a high-molecular-weight polymer made of synthetic rubber, which is made of polyurethane. Polyurethane is a thermosetting polymer formed by diisocyanate’s reaction and polyols’ polymerisation. This polymer is easy to stretch, and internal stress is generated so that it can rebound to its original length after stretching within limits [16]. In the 1970s, a 28-day study reported that most of the force degradation of elastomeric chains occurs on the first day of stretching and then enters a relatively stable state [17]. Many studies have shown that force occurs immediately after the initial stretching and reduces rapidly during the first 24 h, at which 30–50% of the initial force is lost; the relatively flat stage follows, with the force of 28 days showing an increase of approximately 10% only compared with that on the first day [18–20].

It has been established that the force decay of elastomeric chains is influenced by various factors, such as time, manufacturer, prestretching, method of manufacturing, color of elastomeric chains/modules, and the oral environment [21, 22]. Many studies have investigated the effects of temperature on the force degradation of elastomeric chains. To study the force degradation of elastomeric chains, some researchers simulated the oral temperature and compared it with the normal environment [23, 24]. They found that the force degradation increases with temperature at 10 °C, 22 °C, and 37 °C. Some researchers compared the force degradation of elastomeric chains at 60 °C and 37 °C and found that the force degradation in the former is significantly higher than that in the latter [25]. Stevenson et al. [26] tested the tensile properties of elastomeric chains at different temperatures and found that the stress relaxation of elastomeric chains gradually increases as the temperature increases. The mechanical properties of elastic chains are affected by several factors. The intrinsic factors of elastomeric chains, such as the type of filament and the number of loops, also have a certain effect. In addition, the temperature of the oral environment easily changes with food intake. However, the above studies selected only the 37 °C saliva environment, without additional thermal cycling conditions to better simulate the oral environment, and these studies were conducted many years ago. The mechanical properties of elastic chains have improved with the technology advances, requiring the improvement of the experimental conditions to validate the results.

The study's null hypothesis would be that there is no relationship between the filament type, the number of loops, and the force degradation of elastomeric chains under thermal cycling conditions. Therefore, this study investigated the force decay of elastic chains with 250 g of initial force in a 37 °C artificial saliva environment for 28 days at different time points and analyzed the effect of connector types and the number of loops on the force decay of the elastic chain under thermal cycling conditions.

## Methods

### Samples

The study was approved by the ethics committee of the Stomatology Hospital of China Medical University, Liaoning, China (No: CMUKQ-2021-026). All methods were carried out in accordance with the principles of the declaration of Helsinki.

Three elastomeric chains from one company (G&H Orthodontics, 2165 Earlywood Drive, Franklin, IN 46131 USA) were used in this study. Four, five, and six elastomeric chain loops were prepared. According to the orthogonal design, nine groups were formed, namely, the close, medium, and long elastomeric chain groups (A1B1 to A3B3, A1, A2, A3). B1, B2, and B3 are groups of four, five, and six elastomeric chain loops of different elastomeric chains and loops, each containing 15 samples.

### Measurement of force decay

First, five samples from each group were used to measure the stretching length to fabricate a tension-fixing device, where the initial force value is fixed at 250 g, a nail is fixed on a wooden board at point X as a fixed point on one side of the elastomeric chain, and the rubber chain is stretched with a dynamometer until the force value reaches 250 g. Mark the position of the end point of the elastomeric chain at this time as Y, and then stretch the elastomeric chain from point X to point Y each time and measure the force value. The samples from each group were dropped into an artificial saliva environment (0.26% NaH2PO4•H2O, 2.17% NaHPO4•7H2O, 0.9%NaCl) at 37 °C [27] in electrothermal constant temperature water bath (HWS12, Shanghai, China). The artificial saliva was changed every three days. A thermocycler system was set at between 5 and 55 °C for 30 min each time and three times a day with an interval of 4 h. The force of the elastomeric chains was measured at different time points (i.e., 4 h, 24 h, 7 days, 14 days, 21 days, and 28 days) using an Eide Fort digital force gauge (HP-2, Fuji, Japan, accuracy to 0.001kgf).

### Statistical analysis

All quantitative data are depicted as the mean ± standard deviation and were analyzed using SPSS 25.0.

The Kolmogorov-Smirnov test was performed to check the normality of the data. The difference in degradation percentage between the groups was analyzed by multiple factor variance analysis. Significant differences were defined as *p* < 0.05.

## Results

### Remaining force of the elastomeric chains in each group at different time points

The initial force of the elastomeric chains in each group was 250 g. All the forces in the elastomeric chain were gradually reduced in a time-dependent manner. The force decreased significantly in the initial 4 h, and the remaining force dropped to 177.2 ± 5.0 to 204.6 ± 7.7 g in the next 4 h. At 24 h, the remaining force of Groups A1 and A3 was slightly higher and lower than that of the other groups, respectively. The remaining force of all the elastomeric chains dropped to the lowest after 28 days, and the remaining force of Group A3 is the minimum (Table1).

**Table 1:** Remaining force of the elastomeric chains in each group at different time points. (unit: g)

Experimental groups	4 h	24 h	7 days	14 days	21 days	28 days
A_1_B_1_	191.0 ± 7.4	175.6 ± 11.7	168.9 ± 10.1	161.2 ± 7.7	155.0 ± 10.6	147.8 ± 11.3
A_1_B_2_	195.4 ± 6.9	190.0 ± 5.5	186.9 ± 11.4	183.0 ± 8.0	180.7 ± 11.9	175.3 ± 13.4
A_1_B_3_	195.5 ± 7.0	189.6 ± 5.6	186.8 ± 11.6	183.7 ± 8.2	181.6 ± 12.1	177.5 ± 12.8
A_2_B_1_	184.1 ± 6.4	170.7 ± 7.3	161.0 ± 10.2	158.1 ± 5.3	150.1 ± 11.2	140.8 ± 6.5
A_2_B_2_	198.5 ± 8.9	175.3 ± 9.5	170.1 ± 8.3	168.8 ± 9.5	167.8 ± 10.8	167.0 ± 8.8
A_2_B_3_	202.3 ± 9.7	178.8 ± 10.4	170.6 ± 9.1	170.1 ± 10.3	168.3 ± 11.8	167.3 ± 9.6
A_3_B_1_	177.2 ± 5.0	153.5 ± 10.4	141.9 ± 12.8	131.6 ± 9.3	123.9 ± 6.4	115.1 ± 11.8
A_3_B_2_	197.4 ± 7.0	169.2 ± 11.2	156.5 ± 9.6	146.4 ± 7.3	137.6 ± 10.2	130.1 ± 10.8
A_3_B_3_	204.6 ± 7.7	180.0 ± 12.3	167.4 ± 10.6	157.4 ± 8.1	149.2 ± 11.2	142.6 ± 11.9

Values are expressed as mean ± SD. A1, A2, and A3 are the groups of close, medium, and long elastomeric chains, respectively. B1, B2, and B3 represent elastomeric chains of four, five, and six loops, respectively

### Percentage of force degradation on the elastomeric chain in each group was measured at different time points

The percentage of force degradation on the elastomeric chain gradually decreased in a time-dependent manner. The percentage of force degradation at 4 h was approximately 20–35%, while the percentage of force degradation at 24 h was nearly 30–45%. The percentage of force degradation is nearly the same between 24 h and 28 days. At 28 days, the percentage of force degradation was 35–55% (Table 2).

**Table 2:** Percentage of force degradation (%) in each group at different time points

Experimental groups	4 h	24 h	7 days	14 days	21 days	28 days
A_1_B_1_	25.7 ± 2.9	31.7 ± 6.1	34.3 ± 5.8	37.3 ± 4.6	39.7 ± 6.6	42.5 ± 7.3
A_1_B_2_	24.4 ± 2.7	26.5 ± 2.8	27.7 ± 6.0	29.2 ± 4.3	30.1 ± 6.5	32.2 ± 7.4
A_1_B_3_	23.7 ± 2.7	26.0 ± 2.9	27.1 ± 6.1	28.3 ± 4.4	29.1 ± 6.6	30.7 ± 7.0
A_2_B_1_	28.6 ± 2.5	33.8 ± 4.0	37.5 ± 6.0	38.7 ± 3.3	41.8 ± 7.1	45.4 ± 4.3
A_2_B_2_	23.2 ± 3.4	32.2 ± 4.8	34.2 ± 4.7	34.7 ± 5.6	35.1 ± 6.4	35.4 ± 5.2
A_2_B_3_	20.9 ± 3.8	30.1 ± 5.1	33.3 ± 5.1	33.5 ± 6.0	34.2 ± 6.9	34.6 ± 5.7
A_3_B_1_	31.2 ± 1.9	40.4 ± 5.9	44.9 ± 8.3	48.9 ± 6.6	51.9 ± 4.9	55.3 ± 9.5
A_3_B_2_	23.7 ± 2.7	34.6 ± 5.7	39.5 ± 5.7	43.4 ± 4.7	46.8 ± 7.0	49.7 ± 7.8
A_3_B_3_	20.2 ± 3.0	29.8 ± 6.0	34.7 ± 5.9	38.6 ± 4.8	41.8 ± 7.1	44.4 ± 8.0

Values are expressed as mean ± SD. A1, A2, and A3 are the groups of close, medium, and long elastomeric chains, respectively. B1, B2, and B3 represent elastomeric chains of four, five, and six loops, respectively

### Curve of force degradation on different loops of elastomeric chain

**Fig. 1:**
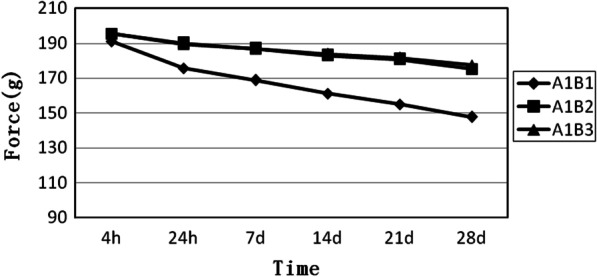
Force decay curves of the close elastomeric chains with four, five, and six loops, respectively. A1B1, close elastomeric chains with 4 loops. A1B2, close elastomeric chains with 5 loops. A1B3, close elastomeric chains with 6 loops

A1 is a group of close elastomeric chains. As shown in Fig. 1, the curve of the four loops is the lowest at the beginning, and the slope of the curve is relatively large. The five- and six-loop groups are relatively straight. The results show that the force degradation decreased slowly as the number of loops increased (Fig. 1).

**Fig. 2:**
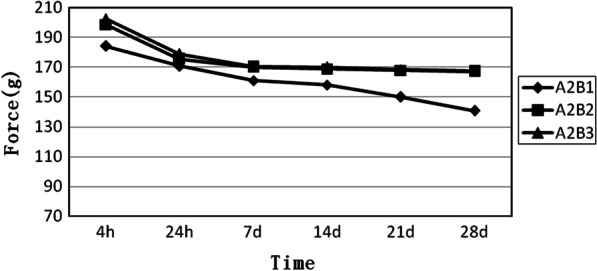
Force decay curves of the medium elastomeric chains with four, five, and six loops, respectively. A2B1, medium elastomeric chains with 4 loops. A2B2, medium elastomeric chains with 5 loops. A2B3, medium elastomeric chains with 6 loops

A2 is a group of medium elastomeric chains. As shown in Fig. 2, the three curves are relatively close to each other. The trends for B2 and B3 are nearly the same, while B1 exhibits the lowest force. The results indicate that the force degradation increases as the number of loops decreases (Fig. 2).

**Fig. 3:**
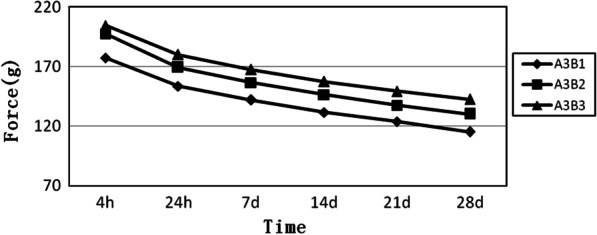
Force decay curves of the long elastomeric chains with four, five, and six loops, respectively. A3B1, long elastomeric chains with 4 loops. A3B2, long elastomeric chains with 5 loops. A3B3, long elastomeric chains with 6 loops

A3 is a group of long elastomeric chains. The slope of the three groups is nearly the same. The group of six loops is at the top, while the group of four loops is at the bottom. The figure shows that the force degradation increases as the number of loops decreases (Fig. 3).

### Force decay curves of elastomeric chains which had different connectors but the same number of loops

**Fig. 4:**
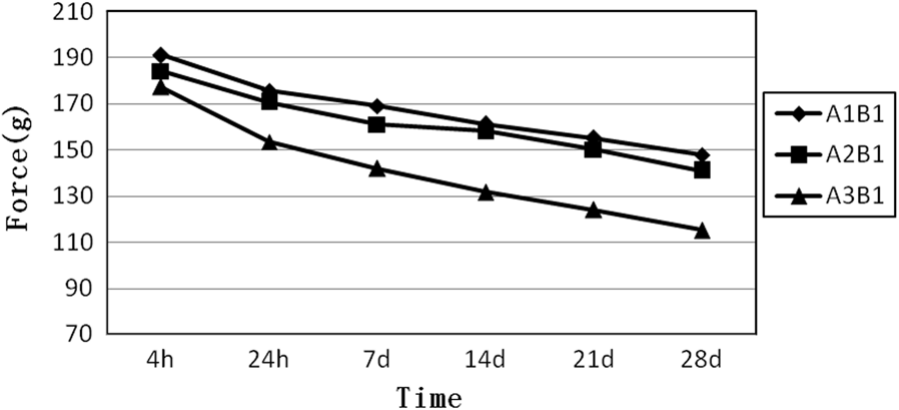
Force decay curves of the elastomeric chains have four loops and different connectors. A1B1, close elastomeric chains with 4 loops. A2B1, medium elastomeric chains with 4 loops. A3B1, long elastomeric chains with 4 loops

B1 is a group of four-loop elastomeric chains. Figure 4 shows that the slopes of the three curves are nearly the same. The close elastomeric chain is at the top, and the long elastomeric chain is at the bottom. The figure shows that the force degradation of the elastomeric chain gradually increases with the increase in the length of the elastomeric chain (Fig. 4).

**Fig. 5:**
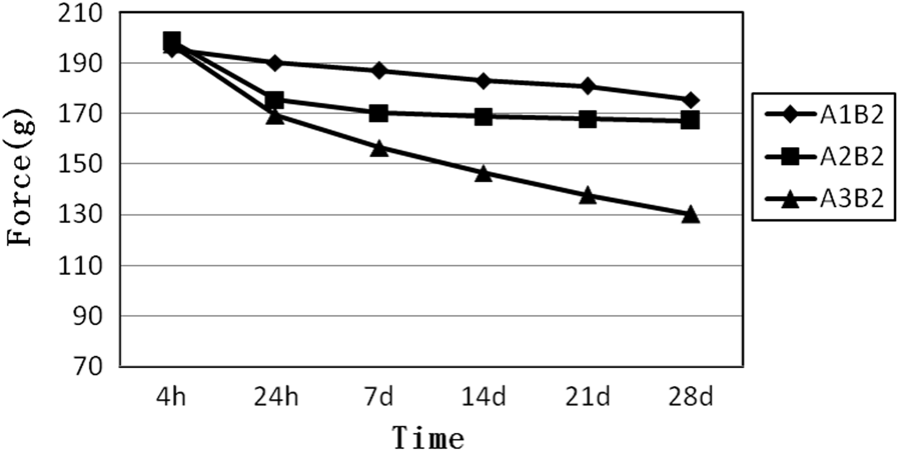
Force decay curves of the elastomeric chains have five loops and different connectors. A1B2, close elastomeric chains with 5 loops. A2B2, medium elastomeric chains with 5 loops. A3B2, long elastomeric chains with 5 loops

B2 is a group of five-loop elastomeric chains. Figure 5 shows that the starting points of the three curves are relatively concentrated and begin to disperse with different slopes. The closed elastomeric chain is at the top, and the long elastomeric chain is at the bottom. As the connector length increases, the force degradation of the elastomeric chain gradually increases(Fig. 5).

**Fig. 6:**
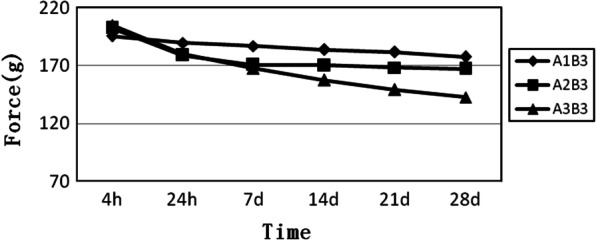
Force decay curves of the elastomeric chains have six loops and different connectors. A1B3, close elastomeric chains with 6 loops. A2B3, medium elastomeric chains with 6 loops. A3B3, long elastomeric chains with 6 loops

B3 is a group of six-loop elastomeric chains. We can see from Fig. 6 that the three curves are relatively concentrated even though they have some intersections. Overall, the close elastomeric chain is at the top, and the long elastomeric chain is at the bottom (Fig. 6).

### Variance analysis results in a significant difference in the connector type and the loops number double factors’ effect on the force decay speed of elastomeric chains

The results show significant differences in the type of elastomeric chains and the percentage of force degradation at different time points (*p* < 0.05). Furthermore, the type of elastomeric chain positively affected the rate of force degradation of the elastomeric chain at different time points, and a significant difference can be observed in the number of loops (*p* < 0.05), which shows that the number of loops affected the rate of force degradation of the elastomeric chains.

### Student–Newman–Keuls (SNK) method analysis results in significant differences in different filament types whose influence on the force decay speed of elastomeric chains

The results show a significant difference between different connector types (*p* < 0.05) at time points. The remaining percentage of force demonstrated when the same initial force was provided is follows: the percentage of degradation in large filaments was the highest, while the percentage of degradation on the six-loop elastic chains decreased the most.

## Discussion

Different brands of elastomeric chains made by diverse manufacturers have dissimilar raw materials and production processes [3]. To eliminate variances in brands, we selected and used one brand of elastomeric chains in this study. In clinical practice, elastomeric chains are mainly used for the distal movement of canine teeth and space closing. The most common position of the elastomeric chain is between the towing hook of the buccal tube of the first molar and the distal canine bracket. Therefore, we could choose the different types and numbers of loops according to individual differences. Four and five loops are most commonly used for the distal movement of canine teeth [28–30]. This study chose four, five, and six loops to provide significant clinical guidance. As the orthodontic patients’ re-examination time is four weeks, this study cycle was conducted for 28 days [3].

There are studies on the effect of different elastomeric chains based on their morphology and elongation extent on force degradation and the ability of elastomeric chains to maintain force over time, as well as how they may affect the overall effectiveness of the appliance in moving teeth [3, 31]. The type of filament and number of loops can affect force degradation, with shorter filaments resulting in slower degradation [31]. As the number of loops increases, the force degradation rate decreases. Other variables include the material used to make the loops [3]; and oral environments, such as the presence of saliva or different types of oral bacteria [1, 5, 7, 32] and color stability [4] on the degradation of elastomeric chains.

Ajmi et al. [33] studied the influence of the type of filament, the number of loops, and the initial force on the mechanical properties of elastomeric chains in an artificial saliva environment at 37 °C. We improved the experiment based on previous studies, adding the thermal cycle condition between 5 and 55 °C to simulate the oral environment. At the same number of loops, the remaining force of the close, medium, and long filament types increased, and the percentage of force degradation of the long, medium, and close filament types increased. Therefore, at the same number of loops, the long elastomeric chain exhibited the highest force degradation. This result is consistent with that of Sang Ting [31]. In addition, Eliades et al. [30] found that the stress of close elastomeric chains is concentrated on the loops and the types with filaments whose stress is distributed on the connector. The longer the connector is, the greater the force degradation will be, probably because the connector is weaker than the loops, and the force degradation is greater in the connector than in the loops. The longer the connector is, the greater the percentage of stress distribution and the force decay are.

In addition, the results show that when the connector types were the same, the remaining forces of the four, five, and six loops increased, while the force degradation decreased in a time-dependent manner. According to Eliades' theoretical analysis, increasing the number of loops in an elastomeric chain weakens the structure, which is prone to decay but also reduces the degradation of the chain [30].

The main component of the elastomeric chain is polyurethane. One part of polyurethane is made of polyether, a spiral chain of adjustable length. Therefore, they can be easily stretched. Stretching the spiral chains into a line changes the structure from random to orderly chains. At this point, the tensile stress is produced in the polymer, and it tends to exhibit a disorderly spiral chain. In the initial stage, the polymer only expands its curly molecule and overcomes the hydrogen-propelled and Van der Waals forces between molecules. Subsequently, the polymer is extended into chain-like molecules, which continue to be stretched to produce a relatively large stress to overcome the covalent bonds between the molecules. In these two phases, the tensile deformation is reversible. When stretching is stopped, the elastomeric chain reverts to its initial formation. When the force continues to increase, the covalent bond between the polymers molecules would be destroyed; the elastomeric chain would produce irreversible, permanent deformation. The limit of the polymer covalent bonds between molecules is called the elastic limit of the elastomeric chain [34].

With constant temperature and deformation, the viscous strain component of the polymer increases, and the rebound strain component decreases in a time-dependent manner, leading to a gradual decrease in the restoring force over time. This phenomenon is called stress relaxation, which is the force degradation discussed in this study. In other words, when the temperature is constant, stretching the elastomeric chain persistently at a constant force within the elastic limit also causes permanent deformation over time, resulting in force degradation [35].

Storie et al. [36] tested the size of elastomeric chains in water and artificial saliva. Their results show that (in water and artificial saliva replacement) both led to dimensional changes in the elastomeric chains. The dimensional change of the elastomeric chains in the water and artificial saliva environments was larger than before. Due to the liquid’s absorption in the environment, the size of the elastomeric chain changed, leading to force degradation.

Canine retraction is an important use of elastomeric chains in clinical applications. Knowing whether the remaining force of various elastomeric chains can still meet the need to move the canine teeth after a while is extremely important. Boester et al. [37] pointed out that when the force exerted on the canine teeth is less than 55 g, it can not cause any physiological reaction in the periodontal tissueHowever, alveolar bone resorption are difficult to induce. Therefore, the body of teeth hardly moves.

The initial force was large in this study, the remaining force of the elastomeric chain at 28 days was relatively large, and the remaining force of each elastomeric chain at a different time point could still meet the need to move the canine teeth. However, under thermal cycle conditions, the force degradation rate of the long elastomeric chain was as high as 55.3 ± 9.5%, suggesting that when using an elastomeric chain with a long connector and few loops, attention should be paid to controlling the initial force. If the initial force is extremely low, a rebound force with clinical significance may not be provided at 28 days. In this situation, the re-examination time can be appropriately shortened.

In addition, due to the variability of the oral environment, such as the fluoride medium, some proteins and microbes in oral saliva also affect elastomeric chains. Therefore, the degree of force degradation differs between the in vitro experiment and the physical truth in the mouth [34].

## Conclusions

According to the present study findings, the null hypothesis was rejected. We concluded that under the initial force, the longer the connecting body is, the fewer the number of loops and the greater the force degradation of the elastomeric chain.

## Data Availability

The datasets generated and analyzed during the current study are not publicly available because of (ownership of data) but are available from the corresponding author upon reasonable request. All data and materials were available at the Stomatology Hospital, China Medical University, Liaoning, China.
